# Association of decreased serum brain-derived neurotrophic factor (BDNF) concentrations in early pregnancy with antepartum depression

**DOI:** 10.1186/s12888-015-0428-7

**Published:** 2015-03-10

**Authors:** Jenny Fung, Bizu Gelaye, Qiu-Yue Zhong, Marta B Rondon, Sixto E Sanchez, Yasmin V Barrios, Karin Hevner, Chunfang Qiu, Michelle A Williams

**Affiliations:** 1 Department of Epidemiology, Harvard T.H. Chan School of Public Health, Boston, MA USA; 2 Department of Medicine, Cayetano Heredia Peruvian University, Lima, Peru; 3 Universidad de Ciencias Aplicadas, Lima, Peru; 4 Asociación Civil PROESA, Lima, Peru; 5 Center for Perinatal Studies, Swedish Medical Center, Seattle, WA USA

**Keywords:** Peru, Biomarker, BDNF, Maternal depression

## Abstract

**Background:**

Antepartum depression is one of the leading causes of maternal morbidity and mortality in the prenatal period. There is accumulating evidence for the role of brain-derived neurotrophic factor (BDNF) in the pathophysiology of depression. The present study examines the extent to which maternal early pregnancy serum BDNF levels are associated with antepartum depression.

**Method:**

A total of 968 women were recruited and interviewed in early pregnancy. Antepartum depression prevalence and symptom severity were assessed using the Patient Health Questionnaire-9 (PHQ-9) scale. Maternal serum BDNF levels were measured using a competitive enzyme-linked immunosorbent assay (ELISA). Logistic regression procedures were performed to estimate odds ratios (OR) and 95% confidence intervals (95% CI) adjusted for confounders.

**Results:**

Maternal early pregnancy serum BDNF levels were significantly lower in women with antepartum depression compared to women without depression (mean ± standard deviation [SD]: 20.78 ± 5.97 vs. 21.85 ± 6.42 ng/ml, p = 0.024). Lower BDNF levels were associated with increased odds of maternal antepartum depression. After adjusting for confounding, women whose serum BDNF levels were in the lowest three quartiles (<17.32 ng/ml) had 1.61-fold increased odds (OR = 1.61, 95% CI: 1.13, 2.30) of antepartum depression as compared with women whose BDNF levels were in the highest quartile (>25.31 ng/ml). There was no evidence of an association of BDNF levels with depression symptom severity.

**Conclusions:**

Lower maternal serum BDNF levels in early pregnancy are associated with antepartum depression. These findings may point toward new therapeutic opportunities and BDNF should be assessed as a potential biomarker for risk prediction and monitoring response to treatment for antepartum depression.

**Electronic supplementary material:**

The online version of this article (doi:10.1186/s12888-015-0428-7) contains supplementary material, which is available to authorized users.

## Background

Antepartum depression is a nonpsychotic depressive episode of mild to moderate severity that presents during pregnancy [1-3]. Studies have shown that the prevalence of depression during pregnancy ranges from 7-15% in high-income countries [2,4] and 19-25% in low- and middle-income countries [5-7]. Antepartum depression is of concern because it has been linked to negative health-related behaviors and outcomes, including poor nutrition, increased substance use, inadequate prenatal care, preeclampsia, preterm delivery, postnatal depression, and suicide [8-16]. Infants delivered of women with untreated antepartum depression are at increased risk of preterm delivery, growth restriction, low birth weight, and other neonatal complications [17-23].

Antepartum depression is a complicated phenomenon which results from the interaction of several psychosocial and neurobiological factors. A number of studies, primarily conducted in men and non-pregnant women, have identified potential biomarkers of depression. One of the most widely studied potential biomarker is the brain-derived neurotrophic factor (BDNF). BDNF is a neurotrophin that is involved in neuronal cell growth, survival, and synaptic plasticity [24]. Early observations by Duman *et al*. indicated that antidepressant drugs increase the synthesis of BDNF and led to the suggestion that a deficiency in neurotrophic factor synthesis and signaling underlie depression and that antidepressants might act by increasing the levels of BDNF and other neurotrophins [25,26]. These observations were later followed by findings supporting a more complex and functional role of BDNF in depression and antidepressant action [25]. Two recent meta-analyses of clinical studies indicate that peripheral blood BDNF levels are decreased in depressed patients before treatment, and that BDNF levels are increased following a course of antidepressant treatment [27,28]. There is accumulating evidence, primarily focused on postpartum period, showing decreased BDNF levels with the presence of depression [29-31]. One of the major risk factors for postpartum depression is antepartum depression [2]. Early detection of antepartum depression is critical in mitigating risks of postpartum depression as well as adverse obstetric outcomes. To add to the emerging literature, we sought to evaluate the extent to which serum BDNF levels are associated with antepartum depression among pregnant Peruvian women.

## Methods

### The PrOMIS Cohort Study

The sample for the present study was drawn from participants of the ongoing Pregnancy Outcomes, Maternal and Infant Study (PrOMIS) Cohort, designed to examine maternal social and behavioral risk factors of preterm birth and other adverse pregnancy outcomes. The study population consists of women attending prenatal care clinics at the Instituto Nacional Materno Perinatal (INMP) in Lima, Peru. The INMP is the primary reference establishment for maternal and perinatal care operated by the Ministry of Health of the Peruvian government. Recruitment began in February 2012. Women eligible for inclusion were those who initiated prenatal care prior to 16 weeks gestation. Women were ineligible if they were younger than 18 years of age, did not speak and read Spanish, or had completed more than 16 weeks gestation.

Enrolled participants were invited to participate in an interview where trained research personnel used a structured questionnaire to elicit information regarding maternal socio-demographic, lifestyle characteristics, medical and reproductive histories, and early life experiences of abuse and with symptoms of mood and anxiety disorders. All participants provided written informed consent. The institutional review boards of the INMP, Lima, Peru and the Harvard T. H. Chan School of Public Health Office of Human Research Administration, Boston, MA approved all procedures used in this study.

### Analytical population

The study population for this report is derived from information collected from those participants who were enrolled in the PrOMIS Study between February 2012 and December 2012. During this period, 1,327 eligible women were approached, and 1,142 (86%) agreed to participate. Of those participating, 982 provided blood samples (86%). Fourteen participants were excluded from the present analysis because of missing information concerning depressive symptoms. Women excluded from this analysis did not differ in regards to sociodemographic and lifestyle characteristics as compared with those included (Additional file 1: Table S1). A total of 968 women remained for analysis.

### Antepartum depression

Antepartum depression was assessed using the Patient Health Questionnaire (PHQ-9) which is a nine-item, self-reported depression measure from the Primary Care Evaluation of Mental Disorders [32,33] diagnostic instrument. The instrument was derived from the criteria of the Diagnostic and Statistical Manual of Mental (DSM-IV) and assessed nine depressive symptoms, namely, anhedonia, depressed mood, trouble sleeping, feeling tired, change in appetite, guilt, or worthlessness, trouble concentrating, feeling slowed down or restless, and suicidal thoughts, experienced over the last two weeks (14 days). The PHQ-9 score was calculated by assigning a score of 0, 1, 2, and 3, to the response categories of “not at all,” “several days,” “more than half the days” and “nearly every day” respectively. As a severity measure, the total score of PHQ-9 ranged from 0 to 27. Depression severity was determined at cut-off scores of 5, 10, 15 and 20, representing the thresholds for none, mild, moderate, moderately severe and severe depression [32]. Possible major depressive disorder (MDD) was defined as total score is 10 or greater [32].

PHQ-9 has been widely used for depression screening in adult primary care setting and hospital settings. It has been translated in to at least 25 languages [34,35] and successfully used to measure depression severity among US African Americans, Asian/Chinese Americans, Latinos, and non-Hispanic Whites [36,37]. Specifically, the Spanish language version of the PHQ-9 was tested and found to be a reliable and efficient screening measure for depression severity in primary care [38-40] and it has been validated among both prenatal and postpartum women.

Following structured interviews, maternal non-fasting blood samples were collected in 7 ml vacutainer tubes containing K3-EDTA (1 mg/ml). Blood samples were kept on wet ice and processed within 20 minutes of phlebotomy. The samples were centrifuged at 850 g for 20 minutes at 4°C. Fractions were aliquoted into cryovials and stored at −80°C until analysis. Serum BDNF levels were measured using a competitive enzyme-linked immunosorbent assay (R&D Systems, Minnesota) with intra- and inter-assay coefficients of variation both <8%. All assays were performed without knowledge of maternal antepartum depression status.

### Statistical analysis

The distribution of maternal socio-demographic characteristics, medical and reproductive histories according to quartiles of maternal serum BDNF levels was examined. Given our large sample, we used visual inspection of quantile-quantile (Q-Q) plot and boxplot to check for normality [41,42]. To estimate the relative association between varying levels of BDNF and odds of antepartum depression, we categorized each subject according to quartiles determined by the distribution of serum BDNF levels among the entire study population. This procedure for determining exposure categories for continuous covariates has been previously described by Hsieh *et al*. [43]. Using the highest quartile as the referent group, odds ratios (OR) and 95% confidence intervals (95% CI) were estimated for each successively lower quartiles. Logistic regression procedures were used to calculate maximum likelihood estimates of odds ratios and 95% confidence intervals, adjusted for potential confounding factors [44]. Confounders were defined as those factors which altered the unadjusted odds ratios by at least 10%. Maternal illicit drug use, smoking and alcohol consumption during pregnancy were found not to be confounders. Maternal age, parity and early pregnancy BMI were considered to be potential confounders. In multivariable logistic regression models, significance for monotonic trends in risk between odds of antepartum depression and serum BDNF levels were assessed by treating the four quartiles as a continuous variable after assigning a score as its value. All reported p values are two sided. All analyses were completed using SAS 9.3 (SAS Institute, Cary, NC, USA).

## Results

Characteristics of study participants distributed according to quartiles of maternal early pregnancy serum BDNF levels are summarized in Table 1. Briefly, women with higher BDNF levels tended to be older, multiparous, heavier and were more likely to report having planned the current pregnancy. Approximately 28.9% of this cohort of pregnant women met the diagnostic criteria for antepartum depression on the basis of their PHQ-9 score: ≥10. As shown in Table 2, depressed women had lower mean serum BDNF levels as compared with non-depressed women (mean ± standard deviation [SD]: 20.78 ± 5.97 vs 21.85 ± 6.42 ng/ml, p = 0.024). As shown in the bottom panel of Table 2, there was no evidence of a linear trend of increasing BDNF levels with increasing severity of depressive symptoms (p = 0.144).

**Table 1:** **Characteristics of study participants according to quartiles of maternal serum brain**-**derived neurotrophic factor** (**BDNF**, **ng**/**mL**) **levels**, **PrOMIS Cohort Study**, **Lima**, **Peru**, **2012**

Characteristics	Serum brain- derived neurotrophic factor (BDNF, ng/ ml)								^ ** ^*P* -value
	Quartile 1		Quartile 2		Quartile 3		Quartile 4		
	(<17.32)		(17.32- 20.92)		(20.92- 25.31)		(>25.31)		
	N = 242		N = 242		N = 242		N = 242		
	n	%	n	%	n	%	n	%	
Maternal age (years)^*^	26.4 ± 5.9		27.3 ± 6.3		28.7 ± 6.0		29.7 ± 6.4		0.001
Maternal age (years)									
18-20	26	10.7	20	8.3	8	3.3	6	2.5	0.091
20-29	151	62.4	142	58.7	132	54.5	121	50.0	
30-34	34	14.0	41	16.9	58	24.0	59	24.4	
≥35	31	12.8	39	16.1	44	18.2	56	23.1	
Gestational age at Interview^*^ (weeks)	10.96 ± 3.5		10.51 ± 3.31		9.6 ± 3.40		8.56 ± 3.11		0.48
Education (years)									
≤6	10	4.1	7	2.9	10	4.2	11	4.5	0.42
7-12	141	58.3	142	59.2	144	60.0	124	51.2	
>12	91	37.6	91	37.9	86	35.8	107	44.2	
Hispanic Ethnicity	179	74.3	186	76.9	180	74.4	186	76.9	0.67
Married/living with partner	190	79.2	194	80.5	192	80.0	201	83.4	0.28
Employed	104	24.2	95	22.1	117	27.3	113	26.3	0.15
Access to basic foods									
Hard	127	52.5	136	56.2	119	49.2	130	53.7	0.82
Not very hard	115	47.5	106	43.8	123	50.8	112	46.3	
Nulliparous	134	55.4	124	51.2	125	51.7	100	41.3	0.004
Planned pregnancy	82	19.7	111	26.6	110	26.4	114	27.3	0.005
Early pregnancy body mass index (kg/m^2^)									
<25	143	59.8	136	56.7	107	44.6	93	38.6	0.026
25-29.9	81	33.9	88	36.7	89	37.1	101	41.9	
≥30	15	6.3	16	6.7	44	18.3	47	19.5	
Illicit drug use during pregnancy	4	1.7	3	1.2	1	0.4	1	0.4	0.10
Smoked during pregnancy	13	5.4	16	6.8	7	2.9	8	3.3	0.09
Consumed alcohol during pregnancy	56	23.3	42	17.4	69	28.5	50	20.7	0.79

Due to missing data, percentages may not add up to 100%.

*Mean ± SD (standard deviation).

**P-value of test of linear trend.

**Table 2:** **Distribution of maternal serum brain**-**derived neurotrophic factor** (**BDNF**, **ng**/**mL**) **levels in early pregnancy according to antepartum depressive symptoms**, **Lima**, **Peru**, **2012**

Depression	N	Mean ± SD	Median [IQR] ^ * ^	*P* -value
Depression				
No (0–9)	688	21.85 ± 6.42	21.22 [17.37-25.85]	0.024**
Yes (≥10)	280	20.78 ± 5.97	20.31 [17.08-24.20]	
Depression severity				
None (0–4)	334	21.99 ± 6.76	21.63 [17.30-26.55]	0.144***
Mild (5–9)	354	21.73 ± 6.09	21.15 [17.63-25.62]	
Moderate (10–14)	158	20.42 ± 6.31	20.17 [16.68-24.03]	
Moderately severe (15–19)	88	21.11 ± 5.82	20.08 [17.20-24.45]	
Severe (20–27)	34	21.67 ± 4.54	21.36 [18.06-24.30]	

*IQR = Interquartile range.

**P-value was calculated using Wilcoxon Rank Sum Test.

***P-value was calculated using Kruskal-Wallis Test.

Multivariate adjusted ORs for maternal antepartum depression in relation to maternal early pregnant BDNF levels are reported in Table 3. The adjusted ORs (95% CI) for antepartum depression are 1.51 (0.99-2.30), 1.66 (1.09-2.52), 1.67 (1.10-2.53), and 1.00 for successive quartiles (lowest to highest quartile), with the highest quartile as the referent group. There was no evidence of a statistically significant linear trend in odds of antepartum depression across decreasing quartile of BDNF (p-value = 0.082). Given the similar magnitude of risks, we collapsed the upper three quartiles and repeated analyses. As shown in the bottom panel of Table 3, compared with BDNF levels in the highest quartile (>25.31 ng/ml), women with lower levels (≤25.31 ng/ml) had a 1.61-fold higher odds of antepartum depression (OR = 1.61; 95% CI: 1.13-2.30) after adjustments for confounding factors including maternal age, parity and early pregnancy body mass index.

**Table 3:** **Odds ratio** (**OR**) **and 95**% **confidence interval** (**CI**) **for maternal serum brain**-**derived neurotrophic factor** (**BDNF**, **ng**/**mL**) **levels in early pregnancy in relation to antepartum depressive symptoms**, **Lima**, **Peru 2012**

BDNF levels (ng/ ml)	Antepartum depression				Unadjusted OR (95% CI)	Adjusted OR (95% CI)*
	Yes (N = 280)		No (N = 688)			
	n	%	N	%		
**Quartile 1** **(<17.32)**	75	26.8	167	24.3	1.68 (1.11, 2.54)	1.51 (0.99, 2.30)
**Quartile 2** **(17.32-** **20.92)**	78	27.9	164	23.8	1.78 (1.18, 2.68)	1.66 (1.09, 2.52)
**Quartile 3** **(20.92-** **25.31)**	76	27.1	166	24.1	1.71 (1.14, 2.59)	1.67 (1.10, 2.53)
**Quartile 4** **(>25.31)**	51	18.2	191	27.8	Reference	Reference
***P*** **-** ***value for linear trend***					0.019	0.082
**Quartile 1–** **3** **(≤25.31)**	229	81.8	497	72.2	1.73 (1.22, 2.44)	1.61 (1.13, 2.30)
**Quartile 4** **(>25.31)**	51	18.2	191	27.8	Reference	Reference

*Adjusted for maternal age, parity, and early pregnancy body mass index.

## Discussion

Overall, we found that pregnant women with lower early pregnancy serum BDNF levels, a well-studied neurotropic peptide, had higher odds of antepartum depression as compared with their counterparts who had higher levels. After adjusting for confounders, women whose serum BDNF levels were in the lowest three quartiles (≤25.31 ng/ml) had 1.61-fold increased odds (OR = 1.61; 95% CI: 1.13, 2.30) of antepartum depression as compared with women whose BDNF levels were in the highest quartile (>25.31 ng/ml).

To the best our knowledge, this is the first large study to examine maternal early pregnancy serum BDNF levels and antepartum depression. Our findings are consistent with most [45-48] though not all [31,49,50] prior studies of BDNF levels and depression among men and non-pregnant women. In their study of adolescents, Pallavi *et al*. noted that treatment naïve adolescents with depression (n = 64) had significantly lower mean serum BDNF levels as compared with healthy controls (n = 64) (8.27 ± 0.39 vs. 10.30 ± 0.39 ng/ml; p < 0.001). The authors also reported that BDNF levels were inversely correlated with Beck’s inventory score (BDI-II) (ρ = −0.32, p < 0.01) [46]. Satomura *et al*., in their study of men and non-pregnant women with major depressive disorder, reported statistically significant inverse associations of serum BDNF level with Hamilton rating scale for depression (HAM-D) score (n = 109, ρ = −0.19, p = 0.044) [47]. Participants classified as depressed on the HAM-D had statistically significantly lower mean BDNF levels as compared with non-depressed participants (20.32 ± 8.18 vs. 27.10 ± 8.31 ng/ml; p < 0.001). Similarly, Karege *et al*. reported decreased mean serum BDNF levels in depressed patients (n = 30) when compared with healthy controls (n = 30) who were matched to cases on sex and age (22.60 ± 3.00 vs. 26.50 ± 7.00 ng/ml; p < 0.001) [45]. Varambally *et al*. found that 43 antidepressant-naive out-patients with depression (assessed using the Mini International Neuropsychiatric Interview) had statistically significantly lower mean serum BDNF level (18.6 ± 4.9 ng/ml) as compared with 24 age-matched healthy controls (23.6 ± 5.6 ng/ml; p < 0.001) [48]. Recently Azevedeo Cardoso and colleagues, in their study of 120 depressed participants and 120 healthy controls (matched for sex, age and tobacco use), noted that mean serum BDNF levels were statistically significantly lower in depressed subjects as compared with controls (4.65 ± 4.18 vs. 7.37 ± 2.31 ng/ml p < 0.001) [51]. On balance, our observation of increased odds of antepartum depression with lower BDNF levels in early pregnancy are consistent with these earlier studies that have principally focused on adolescents, men and non-pregnant women.

Our results, however, are not consistent with findings reported by some other investigators. For instance, Pinheiro *et al*., in their study of 29 depressed and 161 non-depressed Brazilian postpartum women, found no statistically significant differences in mean serum BDNF levels for postpartum depressed and non-depressed women (2.08 ± 1.32 vs. 2.28 ± 1.31 ng/ml, p-value = 0.443) [31]. However, the authors noted that women with postpartum depression and suicidality had statistically significantly lower mean BDNF levels as compared to women without postpartum depression and suicidality (1.50 ± 1.38 vs. 2.32 ± 1.28 ng/ml, p = 0.026) [31]. In their study of 139 participants with major depressive disorder Jevtovic *et al*., found that serum BDNF levels were not significantly associated with severity of depression as assessed by HAM-D (p = 0.169) [49]. Mean serum BDNF levels in patients with mild, moderately severe and severe forms of depression were 41.7 ± 14.5 ng/ml, 36.4 ± 14.8 ng/ml, and 42.1 ± 11.1 ng/ml respectively [49].

The inconsistency of results across studies may be attributable to a number of factors including differences in study populations (e.g., differing underlying distributions treated and un-treated patients), study settings, operational definitions of depression and depressive symptom severity. In addition, the relatively small size of some studies, particularly those failing to document associations of BDNF levels with depression, may have had limited the statistical power for testing their study hypotheses. It is possible that variation in sensitivities of assays used in prior studies may have contributed to variations in reported absolute values of BDNF levels observed across studies; and that variations in assays may have contributed to inconsistent findings. The overall mean BDNF levels, irrespective of depressive symptoms, reported in our study are comparable with most prior studies [45,47,48]. However, the mean BDNF levels in our study are higher than those reported by Azevedeo Cardoso et al. [51] and Pinheiro et al. [31] but lower than Jevtovic et al. [49]. Although a large number of studies have determined serum BDNF levels using ELISA kits, the variability noted in overall mean BDNF levels irrespective of depression status might be reflective of pre-analytical conditions of BDNF assessment [52]. BDNF levels are also known to vary depending on participants’ age, ethnicity, and diet habits [51,53,54].

Observed associations of increased odds of antepartum depression with decreased BDNF is biologically plausible. BDNF, an important and well-studied, neurotrophic factor, has been shown to play a critical role in neurogenesis and neural plasticity [55]. A study using RNA interference and lentiviral vectors to knockdown BDNF expression in specific sub-regions of the hippocampus in mice provoked depressive behaviors and impaired neuronal differentiation [56]. Other animal models have examined factors that influence BDNF expression and levels in peripheral circulation. Franklin and Sinal [57] demonstrated that stress down-regulates BDNF mRNA and protein expression in the hippocampus and other brain regions in rodents. Moreover, administration of synthetic glucocorticoid, dexamethasone, in pigs has been shown to result in decreased BDNF mRNA expression in all hippocampal regions [58]. Collectively, these studies in diverse animal models are consistent with the notion that reduction in BDNF levels may be causally related to depression and depressive symptoms. Of note, in autopsy studies, increased BDNF expression was found in hippocampal regions in subjects with a history of being treated with antidepressant medications as compared with untreated subjects [59,60].

Findings from clinical studies suggest that normalization of BDNF levels in peripheral blood samples may be predictive of antidepressant treatment response [61]. A number of clinical studies have documented increases in serum or plasma BDNF levels after 6 to 8 weeks of treatment among patients who responded to antidepressants, while no similar increases were evident among patients who failed to respond to antidepressants [62-64]. For example, in a small study involving 41 patients with major depression, low serum BDNF levels predicted treatment failure and higher depressive symptomology [63]. On balance, available evidence suggest that low BDNF is predictive of incident [65] and prevalent [46,47] depression and that normalization of BDNF levels may be indicative of treatment of depression [62-64]. Additional studies focused on the relationship of BDNF with the incidence and course of depression during the antepartum and postpartum periods are warranted.

The strengths of our study include a large sample size, the inclusion of antidepressant-naïve study population, use of well-trained interviewers, and rigorous analytic approaches that included accounting for confounding control. However, limitations to our study must also be acknowledged. The cross-sectional design of the present study does not allow for an empirical assessment of the temporal relationship of alterations in BDNF levels and occurrence or onset of antepartum depression. Longitudinal studies with serial blood sampling, determination of BDNF levels, and assessment of depressive symptoms will enhance causal inference in this line of research. Although the PHQ-9 has been validated and has documented to be an efficient screening tool for depression in adult primary care and hospital settings [51,66,67], the possibility of misclassification of depression remains. The impact of potential non-differential misclassification of participants’ depression status in this study is not likely to be related to measured BDNF levels (testing was completed in a blinded manner). Hence, we speculate that any misclassification in maternal antepartum depression status is generally likely to have resulted in an underestimation of the true magnitude of association between lower BDNF levels and antepartum depression risk. The concordance of our study findings with other studies [45-48] that used screening and diagnostic instruments serve to attenuate concerns about misclassification of depression status. Although we used multivariable logistic regression procedures to adjust for putative confounders, we cannot exclude the possibility of residual confounding. Another important limitation that merits consideration is measurement of BDNF levels. Studies have shown that BDNF is first synthesized as a precursor proBDNF, which is proteolytically cleaved to generate mature BDNF [68-70]. Recent studies have demonstrated that proBDNF and mature BDNF facilitate long-term depression and long-term potentiation, respectively, implying opposing cellular functions [69,70]. The ELISA assay used in our study measured the total of BDNF levels (proBDNF and mature BDNF combined), and thus we were not able to assess possible differences of antepartum depression in the relation with proBDNF and mature BDNF. Assessment of proBDNF and mature BDNF levels in future studies is warranted [68].

## Conclusion

Early detection of antepartum depression and effective interventions intended to manage symptoms are critical to prevent adverse perinatal outcomes particularly for socioeconomically disadvantaged women in developing countries and in the United States [4,71]. However, antepartum depression is often under recognized and untreated [72,73]. Currently, depression diagnosis and treatment response relies on interview-based assessments of subjective self-reported symptoms. The results of our study and those of others [45-47] raise the possibility that serum BDNF levels may point toward new therapeutic opportunities, and suggest that BDNF should be evaluated as a possible biomarker for risk prediction and monitoring response to treatment for antepartum depression. Longitudinal data with serial measures of BDNF levels and assessments of depressive disorders during pregnancy and the postpartum periods of pregnancy are warranted.

## Additional file

Additional file 1: Table S1. Characteristics of study participants according to maternal serum brain-derived neurotrophic factor (BDNF, ng/ml) status, PrOMIS Cohort Study, Lima, Peru, 2012.
