# Supplementary material for: Association of decreased serum brain-derived neurotrophic factor (BDNF) concentrations in early pregnancy with antepartum depression
Source: BMC Psychiatry. 2015 Mar 10;15:43. doi: 10.1186/s12888-015-0428-7 (PMC4364091; doi:10.1186/s12888-015-0428-7)
Supplement: Additional file 1: Table S1. — Characteristics of study participants according to maternal serum brain-derived neurotrophic factor (BDNF, ng/ml) status, PrOMIS Cohort Study, Lima, Peru, 2012. [file 12888_2015_428_MOESM1_ESM.doc]

**Additional file 1: Table S**1. Characteristics of study participants according to maternal serum brain-derived neurotrophic factor (BDNF, ng/ml) status, PrOMIS Cohort Study, Lima, Peru, 2012-2014

| **Characteristics** | **Total**  **N=1135** | |  | **BDNF Levels Measured** | | | | |  | ***P*-value** |
| --- | --- | --- | --- | --- | --- | --- | --- | --- | --- | --- |
|  |  |  |  | **Yes**  **N=982** | |  | **No**  **N=153** | |  |  |
|  | **n** | **%** |  | **n** | **%** |  | **n** | **%** |  |  |
| Maternal age (years)* | 28.0 ± 6.2 | |  | 28.0 ± 6.2 | |  | 27.9 ± 5.9 | |  | 0.95 |
| Maternal age (years) |  |  |  |  | |  |  | |  |  |
| 18-20 | 70 | 6.2 |  | 60 | 6.1 |  | 10 | 6.5 |  | 0.82 |
| 20-29 | 641 | 56.5 |  | 556 | 56.6 |  | 85 | 55.6 |  |  |
| 30-34 | 228 | 20.1 |  | 195 | 19.9 |  | 33 | 21.6 |  |  |
| ≥35 | 193 | 17.0 |  | 171 | 17.4 |  | 22 | 14.4 |  |  |
| Gestational age at interview (weeks)* | 9.3 ± 3.5 | |  | 9.3 ± 3.5 | |  | 9.0 ± 3.5 | |  | 0.25 |
| Education (years) |  |  |  |  |  |  |  |  |  |  |
| ≤6 | 49 | 4.3 |  | 39 | 4.0 |  | 10 | 6.5 |  | 0.32 |
| 7-12 | 643 | 56.7 |  | 560 | 57.0 |  | 83 | 54.2 |  |  |
| >12 | 436 | 38.4 |  | 379 | 38.6 |  | 57 | 37.3 |  |  |
| Hispanic ethnicity | 853 | 75.2 |  | 741 | 75.5 |  | 112 | 73.2 |  | 0.92 |
| Married/living with partner | 908 | 80.0 |  | 788 | 80.2 |  | 120 | 78.4 |  | 0.95 |
| Employed | 491 | 43.3 |  | 433 | 44.1 |  | 58 | 37.9 |  | 0.21 |
| Access to basic foods |  |  |  |  |  |  |  |  |  |  |
| Hard | 599 | 52.8 |  | 519 | 52.9 |  | 80 | 52.3 |  | 0.93 |
| Not very hard | 533 | 47.0 |  | 463 | 47.1 |  | 70 | 45.8 |  |  |
| Nulliparous | 568 | 50.0 |  | 489 | 49.8 |  | 79 | 51.6 |  | 0.60 |
| Planned pregnancy | 484 | 42.6 |  | 421 | 42.9 |  | 63 | 41.2 |  | 0.78 |
| Early pregnancy body mass index (kg/m^2^) |  |  |  |  |  |  |  |  |  |  |
| <25 | 567 | 50.0 |  | 486 | 49.5 |  | 81 | 52.9 |  | 0.43 |
| 25-29.9 | 410 | 36.1 |  | 364 | 37.1 |  | 46 | 30.1 |  |  |
| ≥30 | 143 | 12.6 |  | 124 | 12.6 |  | 19 | 12.4 |  |  |
| Illicit drug use during pregnancy | 9 | 0.8 |  | 9 | 0.9 |  | 0 | 0.0 |  | 0.61 |
| Smoked during pregnancy | 50 | 4.4 |  | 45 | 4.6 |  | 5 | 3.3 |  | 0.49 |
| Alcohol consuming during pregnancy | 256 | 22.6 |  | 219 | 22.3 |  | 37 | 24.2 |  | 0.51 |

Due to missing data, percentages may not add up to 100%; * Mean ± SD (standard deviation); *P-*value from Kruskal-Wallis test, Chi-square test or Fisher exact test
